# Pathogenesis, imaging and clinical characteristics of CF and non-CF bronchiectasis

**DOI:** 10.1186/s12890-018-0630-8

**Published:** 2018-05-22

**Authors:** Jürgen Schäfer, Matthias Griese, Ravishankar Chandrasekaran, Sanjay H. Chotirmall, Dominik Hartl

**Affiliations:** 1 Department of Radiology, Division of Pediatric Radiology, University of Tübingen, Tübingen, Germany; 2 Children’s Hospital, University of Munich, Munich, Germany; 3 Lee Kong Chian School of Medicine, Nanyang Technological University, Singapore, Singapore; 4 Department of Pediatrics I, University of Tübingen, Tübingen, Germany; 5 Roche Pharma Research & Early Development (pRED), Immunology, Inflammation and Infectious Diseases (I3) Discovery and Translational Area, Roche Innovation Center, Basel, Switzerland

## Abstract

Bronchiectasis is a common feature of severe inherited and acquired pulmonary disease conditions. Among inherited diseases, cystic fibrosis (CF) is the major disorder associated with bronchiectasis, while acquired conditions frequently featuring bronchiectasis include post-infective bronchiectasis and chronic obstructive pulmonary disease (COPD). Mechanistically, bronchiectasis is driven by a complex interplay of inflammation and infection with neutrophilic inflammation playing a predominant role. The clinical characterization and management of bronchiectasis should involve a precise diagnostic workup, tailored therapeutic strategies and pulmonary imaging that has become an essential tool for the diagnosis and follow-up of bronchiectasis. Prospective future studies are required to optimize the diagnostic and therapeutic management of bronchiectasis, particularly in heterogeneous non-CF bronchiectasis populations.

## Background

Bronchiectasis is a condition in which an area of the bronchial lumen is permanently and abnormally widened, with accompanying infection. Bronchiectasis is found in a variety of pulmonary diseases, both genetically caused and acquired, such as severe pulmonary infections and cystic fibrosis (CF), but is also a feature of Kartagener syndrome, chronic obstructive pulmonary diseases (COPD), alpha 1-antitrypsin deficiency, asthma, or primary immunodeficiencies [1–3]. Bronchietasis is caused by long-term excessive inflammatory damage to the airways, which results in tissue breakdown, enlargement of the affected airways and the key clinical symptoms of chronic productive cough and shortness of breath. Globally, in up to half of all cases the cause cannot be identified (idiopathic). Those cases together with several other known aetiologies such as post-infectious and allergic hypersensitivity collectively fall under the category ‘non-cystic fibrosis’ (non-CF) bronchiectasis [4]. Here we discuss the key features of both CF and non-CF related bronchiectasis with respect to their pathogenesis, imaging and clinical management.

## Pathogenesis of bronchiectasis formation

Bronchiectasis mechanistically results from chronic inflammatory microenvironments that trigger airway tissue breakdown. In both CF and non-CF bronchiectasis, the complex interplay between infection and inflammation feeds a pro-inflammatory vicious circle that progressively drives the generation of bronchiectasis and the destruction of the pulmonary architecture [5]. Inflammatory immune cells (mainly activated macrophages and neutrophils) represent the major infiltrating population in disease conditions associated with bronchiectasis and contribute significantly to tissue damage and bronchiectasis generation through the release of their harmful cellular ingredients. Particularly, cell-derived proteases and reactive oxygen species represent key mediators in the degradation and destruction of extracellular pulmonary tissue components, leading to bronchiectasis formation. The precise early immune-mediated mechanisms that trigger and maintain the formation of bronchiectasis remain yet incompletely understood. Regulated immune homeostasis seems to be essential since both immune deficiencies as well as hyper-active immune responses are associated with bronchiectasis. Particularly, the protease-antiprotease imbalance [6, 7], as found in CF and COPD airways, is considered as key pathogenic component in degrading extracellular matrix. Mutations in the cystic fibrosis transmembrane conductance regulator (CFTR) gene are causative for CF lung disease and drive the earliest pathogenic events in epithelial cells that ultimately lead to the genesis of bronchiectasis. Also beyond CF lung disease, CFTR-related cellular mechanisms regulating mucociliary clearance have been involved in cigarette smoke-induced COPD [8].

In the following two sections, we will focus on the microbiological (a) and immunological/inflammatory (b) findings associated with the pathogenesis of bronchiectasis.

### Microbiology

*Pseudomonas aeruginosa* is a common and dominant pathogen found in the airways of both CF and non-CF bronchiectasis patients [9–13]. Chronic infection has been associated with more severe decline in lung function [14–19], increased hospitalizations [20, 21], frequent exacerbations [22] and disease severity [23, 24]. Although clinical manifestations between the two settings vary, their core airway microbiota is largely analogous [25]. Along with *Pseudomonas*, bacteria belonging to other genera such as *Haemophilus, Streptococcus, Staphylococcus*, *Veillonella, Prevotella* and *Achromobacter* also make up the core microbiota observed in bronchiectasis [9, 26, 27]. Interestingly, *P. aeruginosa* and *H. influenzae* have been described to competitively inhibit each other, which in turn alters the core microbiota in the non-CF bronchiectasis airway [28]. Non-tuberculous mycobacteria (NTM) form another significant group of pathogens colonizing CF and non-CF airways [29–31]. *Mycobacterium avium complex* (MAC) and *Mycobacterium abscessus* are most frequently isolated in CF [32, 33] with high rates of multi-drug resistance in these species making them notoriously difficult to treat [34]. NTM belonging to the MAC group are also highly prevalent in non-CF bronchiectasis with a female preponderance [35, 36]. This group of organisms are surprisingly poorly associated with disease severity and exacerbations in the non-CF setting when compared to *Pseudomonas* [37, 38]. In contrast for CF patients, MAC and *M. abscessus* are often associated with an aggressive and accelerated lung function decline [39–42]. Interestingly, bacterial populations do not drastically change between stable and exacerbation states in bronchiectasis. However, viral load has been positively correlated with exacerbations in both CF and non-CF bronchiectasis patients. Infection with viruses belonging to the *coronavirus*, *rhinovirus* and *influenza A/B* virus families are frequently detected during exacerbations of bronchiectasis [43–45]. Whether the occurrence of such viruses, forming part of the airway ‘virome’ in bronchiectasis, are a cause or consequence of exacerbations remains to be elucidated [43, 46]. Most attention towards understanding the microbiome in bronchiectasis is directed at the bacteriome. Although fungi are frequently isolated from the same airways, the role of the pulmonary mycobiome in the pathogenesis of these disease states remains largely elusive [47–49]. Filamentous fungi belonging to the genus *Aspergillus* are frequently isolated fungal organisms in sputum samples from CF patients [50, 51]. Among the different species of *Aspergillus*, *A. fumigatus* is the most common chronic colonizer in CF [47, 52] Allergic bronchopulmonary aspergillosis (ABPA), an allergic *Aspergillus-*associated disease, is a frequent co-morbidity in CF [53], while *Aspergillus* colonization and sensitization has also been independently correlated with lung function declines and radiological severity in CF [54–56]. Only a single study to date has shown that fungi belonging to the *Aspergillus spp.* and *Candida albicans* are also identifiable in the airways of non-CF bronchiectasis patients [57]. Importantly, in a study of severe asthma patients, *Aspergillus* fumigatus sensitization has also been associated with poorer lung function and an increased incidence of bronchiectasis, a likely cause and consequence for this anatomical airway distortion [58, 59]. Among yeasts, *Candida spp.* are frequent colonizers of the bronchiectatic airways [47, 57, 60]. Isolation of *Candida albicans* from such airways is shown to be a predictor for frequent hospital-exacerbations and declines in lung function [61]. Compared to bacteria, our present understanding of fungal pathogenesis in the context of both CF and non-CF bronchiectasis remains limited and further work is required to determine their prevalence, colonization frequency, host-pathogen interaction and risk factor profile in this key patient group.

### Immunology & inflammation

Neutrophil-dominant inflammation is a key feature of bronchiectasis. Sputum neutrophils are higher in bronchiectasis patients versus healthy controls and this correlates with an increased disease severity [62–64]. Both interleukin-8 (IL-8) and leukotriene-B4 (LTB4) are key chemo-attractants required for migration and infiltration of neutrophils into bronchiectatic airways [65]. High systemic IL-8 levels are detectable in individuals with bronchiectasis [66–68]. Antibacterial neutrophil responses (such as reactive oxygen species (ROS) formation) are activated through the IL-8-CXCR1 axis, but proteolytic cleavage mediated by neutrophil elastase (NE), which itself is associated with exacerbations and lung function decline in bronchiectasis, impairs antibacterial neutrophil functions [69, 70]. Uncontrolled NE activity, as found in CF airways, causes further respiratory tissue damage through degradation of extracellular proteins (such as surfactant proteins [71–73]) and cellular surface receptors (such as complement receptors [74]); high NE levels correlating with disease severity and poorer lung function are described in both CF and non-CF bronchiectasis settings [75, 76]. In this context, CXCR receptor antagonists are hypothesized to inhibit neutrophil airway influx and have been shown to be effective in modulating the inflammatory state in bronchiectasis [77, 78]. Airway neutrophils in CF illustrate an impaired phagocytic ability [79]. This is in line with the observation that CF neutrophils have an impaired ROS production, a critical mediator of antimicrobial host defense [80]. Neutrophils defective in their oxidative abilities obtained from non-CF bronchiectasis patients were poorer at bacterial killing when compared to those of healthy controls [81]. Serine proteases are also important neutrophil derived products, released in response to TNF-α signalling. They degrade proteoglycans in the respiratory epithelium subsequently inducing airway damage [82]. In bronchiectasis, activated airway neutrophils secrete an abundance of human neutrophil peptides (HNPs), which have been described to inhibit their phagocytic ability. Importantly, high concentrations of HNPs are detected in both CF and non-CF airways, which in turn may contribute to the decreased phagocytic abilities and higher rates of infection described in both conditions [83]. Poorer clearance of neutrophils by alveolar macrophages further augments the inflammatory state in bronchiectasis [63]. Eosinophils contribute to tissue injury in CF and the presence of eosinophil cationic protein (ECP) heralds the cell activation state. ECP levels are elevated both in the airway and systemically in bronchiectasis [84–86]. Other eosinophilic markers including eosinophil protein X and peroxidase follow a similar pattern and like ECP contribute to poorer pulmonary function [87]. Importantly, eosinophilic granule release in CF may be triggered by NE illustrating the cross-granulocyte talk that occurs in the setting of bronchiectasis [88]. T-cells constitute another key component of the inflammatory response in bronchiectasis [89]. In CF, high T-helper 2 (Th2) [90, 91] and Th17 [91] responses are observed. Th2 cytokines such as IL-4, − 13 and TARC/CCL17 are correlated with a decreased pulmonary function in CF-*Pseudomonas-*colonized patients. Th17 cells, neutrophils and NKT cells are found in abundance in all-cause bronchiectasis compared to healthy controls [92]. While high Th17 infiltrates independently associate with poorer lung function in CF [93], activation of Th17 antigen-specific pathways have been described in non-CF bronchiectasis [94]. IL-17, a central mediator of the Th17 pathway lacks correlation with bronchiectasis disease phenotypes suggestive of the more prominent role that neutrophil-mediated inflammation likely plays in the pathogenesis of bronchiectasis [94]. Both CD8+ T cells and NKT that express pro-inflammatory IFN-γ and TNF-α have been described in paediatric bronchiectasis [95]. Common pro-inflammatory markers such as TNF-α, IL-8, NE and matrix metalloproteinases − 2, − 8 and − 9 (MMP2, MMP8 and MMP9), are all elevated in bronchiectasis with the latter two indicative of a poorer prognostic outcome [96–100]. A seminal study in CF children identified the key risk factors for bronchiectasis: Sly et al. (2013) showed that elevated airway neutrophil elastase activity was major risk factor and predicted bronchiectasis development [101, 102]. Bacterial load in non-CF bronchiectasis has been correlated with increases in airway (NE, IL-8, IL-1β and TNF-α) and systemic (ICAM-1, E-selectin) derived inflammatory markers, phenomena confirmed in vitro using bronchial epithelial cell lines treated with sputum from bronchiectasis patients [103, 104]. Exacerbations of both CF and non-CF bronchiectasis increases inflammation irrespective of bacterial, viral or fungal causation [43, 105, 106]. Interestingly, sTREM-1 a novel inflammatory marker described in a variety of pulmonary disease states including COPD, has also been identified in children with both CF- and HIV-related bronchiectasis although concentrations in the latter setting are highest. High sTREM-1 levels correlate closely with lung function decline and future studies should explore sTREM-1 levels in bronchiectasis of other aetiologies to better understand its role in the pathogenesis of bronchiectasis [107]. Vitamin-D deficiency, observed in CF [108, 109] is associated with increased bacterial infection, exacerbations and poorer lung function [110–112]. This is corroborated in non-CF bronchiectasis where it indicates disease severity and associates with more infection, bacterial colonization, airway inflammation and consequently frequent exacerbations [113].

## Clinical characteristics and management of bronchiectasis

Bronchietasis patients are clinically characterized by sputum production (upon exercise or spontaneously) leading to productive coughing with mucopurulent masses of yellowish, greenish or brown sputum in the morning or over the day. However, bronchiectasis are mainly detected at time points when irreversible structural damage has already been done to the airway architecture. Bronchiectasis initially may be reversible in children, later probably not. Major genetic diseases associated with bronchiectasis include CF, primary ciliary dyskinesia (PCD, Kartagener syndrome), alpha 1-antitrypsin deficiency, primary immunodeficiencies or other rare disorders like Williams-Campbell syndrome and Marfan syndrome. Major acquired causes are severe bacterial infections (*Tuberculosis, Staphylococcus, Klebsiella* and others) or postinfectious bronchiolitis obliterans. Notably, also fungal infections can lead to bronchiectasis, particularly ABPA, as a chronic Th2-driven *Aspergillus fumigatus*-caused pulmonary condition. Based on this, it is essential in the clinical work-up of patients featuring bronchiectasis to screen for these congenital and acquired conditions in order to tailor appropriate treatments and to attenuate disease progression. In a preventive manner, it is key in the above mentioned conditions to diagnose and monitor for pulmonary symptoms and structural changes (using pulmonary function testing and high-resolution computed tomography, HRCT) to avoid established bronchiectasis-related disease. To this end, it is helpful to follow a concept that has been introduced previously to classify forms of bronchitis in children [114–116]. An acute bronchitis, usually triggered by a viral infection, resolves within days or one to two weeks. Sometimes – for many reasons of which most are unknown – symptoms do not resolve spontaneously, but persist. This state is called protracted bacterial bronchitis (PBB). While PBB has been initially established for pediatrics, current publications have discussed and transferred this concept to adults {Birring, 2015 #16382;Gibson, 2010 #16381;Martin, 2015 #16380}. PBB is further differentiated into various forms, depending on the tools used to diagnose it [114, 115]. PBB can be further characterized based on different stratifiers:

- PBB-microbiologic (“PBB-micro”): (1) presence of chronic wet cough (> 4 weeks), (2) respiratory bacterial pathogens growing in sputum or BAL at density of a single bacterial specifies > 10^4^ colony-forming units/ml, and (3) cough resolves following a 2-week course of an appropriate oral antibiotic (usually amoxicillin-clavulanate)
- PBB-clinical: (1) presence of chronic wet cough (> 4 weeks), (2) absence of symptoms or signs of other causes of wet or productive cough, (3) cough resolves following a 2-week course of an appropriate oral antibiotic (usually amoxicillin-clavulanate)
- PBB-extended: as above, but cough resolves only after 4 weeks of antibiotics
- PBB-recurrent: > 3 episodes of PBB per year

**Fig. 1:**
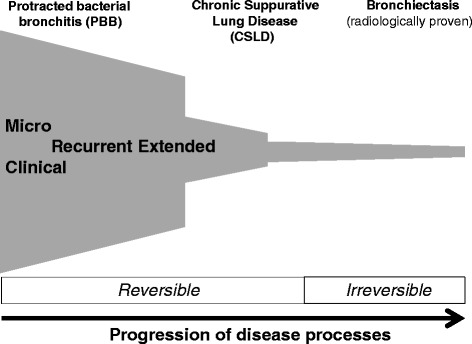
Model of disease progress towards bronchiectasis in patients with and without CF. Modified from Chang et al. [115]

Based on this concept, it is believed that, if left untreated, a fraction of PBBs will progress to chronic suppurative lung disease (CSLD) with radiologically confirmed bronchiectasis (Fig. 1). CSLD differs from bronchiectasis only by lacking the radiographic signs of bronchiectasis on HRCT scans. Clinically, CSLD is diagnosed in children whose chronic wet cough does not resolve with oral antibiotics and in whom other causes are excluded [117, 118]. Although not proven formally for all causes of bronchiectasis, the sequence of progression from PBB over CSLD to bronchiectasis is highly likely, but needs to be substantiated with prospective studies. Of interest is the recent finding that otherwise healthy children with PBB, children with bronchiectasis, and children with CF shared similar core airway microbiota patterns, with *H. influenzae* making the greatest contribution to the observed similarity, while the microbiota in adults with CF and bronchiectasis were significantly different [25]. The authors concluded that chronic airway infections starts similarly with defective airway clearance, but over time with intervention and host factors, i.e. the underlying cause, progressively diverge.

The prevalence of bronchiectasis in children with CF has recently been assessed in studies conducted by the Australian Respiratory Early Surveillance Team for Cystic Fibrosis (AREST CF) and others. Although 50–70% of CF patients have CT-defined bronchiectasis by 3 to 5 years of age [119], most young children have isolated, i.e. localized disease, with only the mildest severity of lung abnormalities and lobar disease extent that is well below 50% [120–122]. On the other hand, it is clear that once established, bronchiectasis persists and/or progresses despite current optimized standard-of-care therapies in about 75% of young children [121, 122]. Currently great effort is undertaken to close the diagnostic gap from 0 to about 5 years of age, to non-invasively assess the extent of lung disease. The PRAGMA-CF score was developed as a sensitive and reproducible outcome measure for assessing the extent of lung disease in very young children with CF [123]. Moreover, the lung clearance index is a measure of ventilation distribution obtained by the multiple-breath washout technique. Several studies have shown its sensitivity to airway disease in CF and other bronchial diseases [124, 125]. However, in infants with CF, lung clearance index was insensitive to structural disease, as assessed by PRAGMA scoring [126]. In preschool and school-age children with CF’s lung clearance index correlated with total disease extent. Of interest, it had good positive predictive value of about 85%, but a poor negative predictive value of 55% to detect bronchiectasis. Therefore, lung clearance index may be a good surveillance tool to monitor structural lung disease up to school-age in CF [126]. In an effort to identify the preceding-stages of bronchiectasis in CF children by using at least four consecutive biennial volumetric CTs, areas with bronchiectasis on CT scans were marked, further analyzed and associated to potential pre-stages, which were mucus plugging (18%), airway wall thickening (2%) or atelectasis/consolidation in 1% [127].

The basic clinical management of bronchiectasis includes tailored antimicrobial therapy and airway clearance techniques. The latter include mucolytics, such as hypertonic saline and rhDNA, as well as chest physiotherapy and vigorous physical sporting activities. In PBB, oral antibiotics for 2 weeks up to several months have been described to be helpful. Antibiotics commonly used in the clinics include amoxicillin, amoxicillin-clavulanate or second generation cephalosporins. Particularly in patients with CF, gram-negative organisms are treated with inhaled tobramycin, colistin, actreonam or levofloxacin as well as oral inhibitors of gyrases, i.e. ciprofloxacin. The duration of treatment should be guided by symptoms; goal is a symptom-free patient. This can be mostly achieved in young children or patients in the stages of PBB, CSLD and the early stages of bronchiectasis. More specific treatment strategies in bronchiectasis depend on the underlying etiology and include protein augmentation (alpha 1-antitrypsin deficiency), anti-allergic approaches (asthma/ABPA) and/or immunoglobulin substitution (immunodeficiencies).

## Imaging of bronchiectasis in CF lung disease

Detection and characterization of bronchiectasis are the domain of thin-section computed tomography (CT). High-resolution CT (HRCT) with 0.6 to 1.5 mm slice thickness serves as a reference standard for imaging. However, pulmonary MRI has gained interest due to the possibility of functional imaging without radiation burden. Moreover, new technical developments overcome the limitations of low MR-signal and low spatial resolution. In CF, standardized reporting using scores or automated quantification are essential requisites to measure and track findings, particularly when results focus on risk stratification. In this context, bronchiectasis is one of the important imaging markers and generally correlates with clinical outcome.

### Imaging characteristics of bronchiectasis

**Fig. 2:**
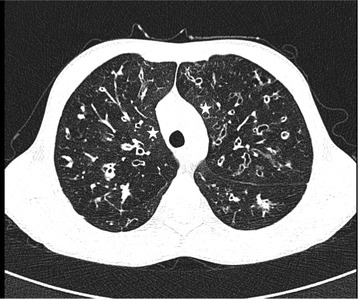
15 y/o male, CF-patient, FEV_1_ predicted 45%. Thin-section HR-reconstruction from MDCT (effective dose of 1.5 mSv). Severe bronchiectasis is visible. Note also dilated bronchi within the periphery of the lung. Air trapping is noted, only in central parenchyma, the CT attenuation appears normal (asterisks)

Bronchiectasis is defined as irreversible dilation of bronchi in cylindrical, varicose, or a more cystic morphological appearance. In CF, it is often associated with mucus plugging, bronchial wall thickening, and small airway disease [128, 129]. The radiological evaluation of bronchiectasis is based on definition published in the terms for thoracic imaging of the Fleischner Society [130]: “*Morphologic criteria on thin-section CT scans include bronchial dilatation with respect to the accompanying pulmonary artery (signet ring sign), lack of tapering of bronchi, and identification of bronchi within 1 cm of the pleural surface*.” The so-called signet ring sign is the primary sign for bronchiectasis representing a ring-shaped opacity, whereas the smaller adjacent artery stays for the signet. According to this concept, the extent of bronchial dilation can be quantified using the ratio between bronchi and vessels [128], an approach challenged by a recent pediatric study [131]. On HRCT, the bronchial tree is only visible up to the 6-8th generation [130]. CT findings like the tree-in-bud sign and centrilobular opacity are linked to small airway disease with dilation and inflammation of the ronchiole or mucus plugging in its periphery (Fig. 2) [130]. There are differences in CF bronchiectasis depending on pancreas insufficiency (PI), with PI patients illustrating more severe bronchiectasis [132]. Primary ciliary dyskinesia (PCD) patients have similar CT scores as pancreatic-sufficient (PS) CF patients, but in contrast to CF, no correlation between structural change and clinical parameters has been detected in a previous study [133]. However, recent studies in adult PCD patient cohorts indicate that CT findings relate to lung function changes [134, 135]. There are no clear identifiers of pre-bronchiectasis in imaging. However, mucus plugging has been shown to be a common precursor in CF [127].

**Fig. 3:**
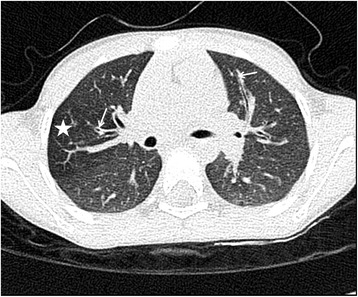
6 y/o female CF-patient, FEV_1_ predicted 105%. Thin-section HR-reconstruction from MDCT (effective dose of 1 mSv). Mild bronchiectasis, bronchial wall thickening (arrows) and mosaic attenuation (asterisk) are visible

**Fig. 4:**
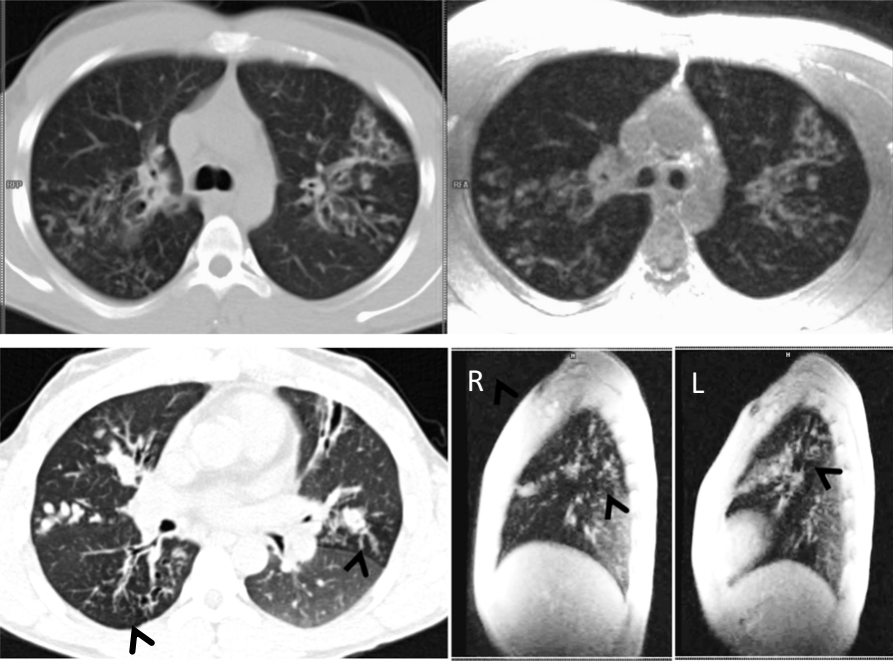
29 y/o male CF-patient, FEV1 predicted 67%. Left side CT, right side MRI at the same day. Upper row, transverse thin-section images from 3D acquisition in breath hold (CT and MRI). Note, despite lower resolution and signal to noise a similar depiction of bronchiectasis is possible. Lower row, expiration images (transverse CT and saggital MRI). In both modalities focal air trapping within the same lung region is demonstrated (arrow heads)

**Fig. 5:**
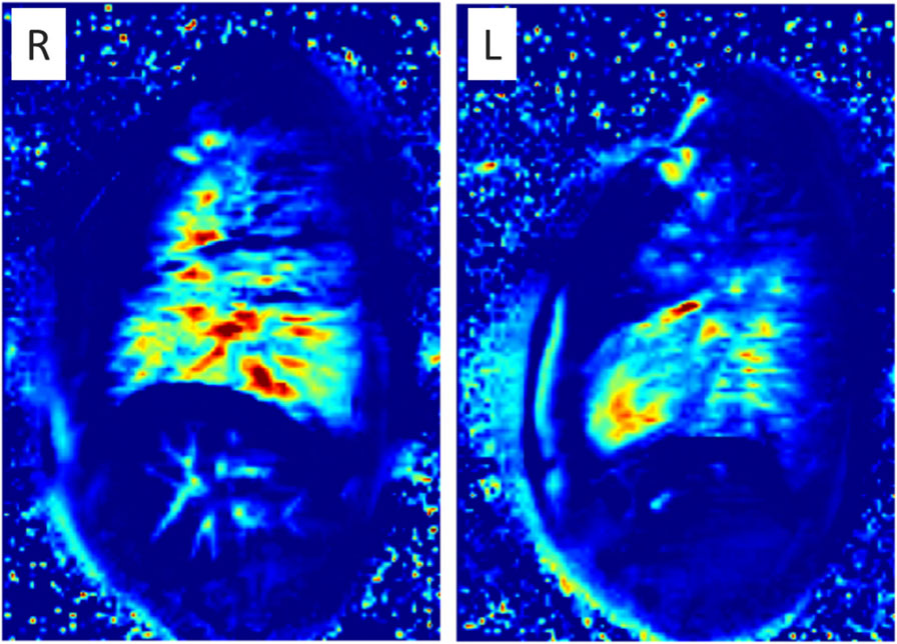
Perfusion map of same patient as in fig. 4 using the noninvasive arterial-spin-labeling technique without contrast media application. The relevant perfusion differences between upper and lower lung regions correlate with morphological damage and air trapping

Imaging can illustrate lung damage even when lung function (such as forced expiratory volume in 1 s, FEV1) is normal [128, 136, 137] (Fig. 3). In contrast to imaging, pulmonary function tests (PFT) are challenging in young children. A complementary role with lung clearance index (LCI) has been described [138]. Regarding the evaluation of the presence and extent of bronchiectasis, CT imaging is accepted as the most sensitive and reproducible modality to date. Using new generation dual source multidetector CT (MDCT) maintaining sub-second acquisition of the whole lung breathing and pulsation artifacts are negligible even in young children and no sedation is needed [139, 140]. Finally, using spectral beam shaping and iterative reconstruction algorithms, ultralow-dose pediatric chest CT can be realized with an effective dose below 0.3 mSv [139]. These conditions, therefore, challenge the routine use of MRI. On the other hand, there are a couple of reasons supporting MRI. The radiation burden of routinely performed volumetric chest CT may be many times higher than that of a third generation of dual source CT recently published. Incremental HRCT with significant gaps lowers the dose but also diagnostic performance and leads to more motion artifact in the pediatric population [141]. Estimated risk of radiation-induced cancer from a pediatric chest CT is small but not negligible, particularly in cases of repeated exposure [142, 143]. MRI has no side effects by radiation enabling long-term surveillance of lung damage. The overall diagnostic performance by scores evaluation of MRI in direct comparison to CT is good to excellent [144–146] (Fig. 4). Moreover, apart from polarized 3+ Helium imaging, functional imaging can be easily implemented using perfusion or ventilation weighted standard proton MRI that assesses small airway disease [146–149] (Fig. 5).

### Clinical value

Standardized reporting of cross-sectional imaging by scoring systems is suitable for several reasons: (a) to assess and to quantify individual progression of lung damage in comparison or complementary to lung function testing, (b) to use total or partial score values as endpoints for interventional studies, and (c) to establish predictive imaging biomarkers. Most CT scoring systems use a semiquantitative scale for the extent and severity of specific findings either based on lobe, involved bronchopulmonary segments, or using an overlay grid [128, 129, 150]. According to the disease-specific prevalence from the imaging abnormalities, sub-scores for bronchiectasis and bronchial wall thickening are more heavily weighted (1, 2). Inter-observer and intra-observer agreement of the common CT scores has shown to be good to excellent [128, 129]. Comparable reproducibility has also been found for MRI using CT or MR-specific adapted scoring systems in small numbers of studies [144, 148]. For semiautomatic evaluation of bronchi dimension excellent inter-observer agreement has been found particularly for the bronchial lumen [151]. On the other hand, mucus can obscure or mimic bronchial wall thickening. Apart from reproducibility, the weighting of abnormalities within the scores, and validation are even more challenging and related to the purpose and the use of score (e.g. interventional or clinical study). As aforementioned imaging is more sensitive than FEV1 particularly in mild disease, and, in assessment of disease progression [128, 136, 137, 152]. In this context scoring of bronchiectasis particularly in the lung periphery is important [137], whereas air trapping, mosaic perfusion, and mucus plugging seem to be more sensitive markers than CT or MRI which detect effects of interventions [153, 154]. The role of bronchiectasis as a robust predictive marker has been shown in several longitudinal observations [150, 155–159]. The extent of bronchiectasis at baseline can predict the number of respiratory tract exacerbations (RTE) [155–158], and the change of the subscore in a two year follow up is strongly associated with numbers of RTEs where FEV1 did not provide value [156]. This has been similarly described for a decade long observational study [158]. In an older study, a maximum combined score for bronchiectasis and emphysema on HRCT was indicative of a poorer prognosis [159]. In a recent study of patients with severe lung disease awaiting lung transplantation, the combined score of bronchiectasis, bronchial wall thickening, mucus, and consolidation was associated with mortality [150].

## Conclusions

Bronchiectasis is a heterogeneous and complex condition and remains a challenge for both diagnostic and therapeutic strategies. While pathomechanisms in the pulmonary compartment share commonalities from a microbiological and immunological perspective, clinical implications and treatment approaches remain challenging and individualized, depending on the underlying disease and the infection status. High-resolution imaging has revolutionized the diagnosis and monitoring of bronchiectasis and will further pave the way for a more precise understanding of disease pathogenesis and treatment response in the future. Therapeutically, lessons learned from the well-known phenotype of CF bronchiectasis are increasingly transferred to the multifaceted genotype and phenotype of non-CF bronchiectasis. Airway infections are treated with inhaled and systemic antibiotics. Mucus clearance can be improved by inhaled therapies and chest physiotherapy, whereas specific anti-inflammatory approaches have still not been clinically established. Prospective future studies are urgently needed to optimize the diagnostic and therapeutic management of bronchiectasis, particularly in children with non-CF bronchiectasis, an indication with a high unmet medical need.

## Abbreviations

ABPA: Allergic bronchopulmonary aspergillosis
CF: Cystic fibrosis
COPD: Chronic obstructive pulmonary disease
CSLD: Chronic suppurative lung disease
ECP: Eosinophil cationic protein
FEV1: Forced expiratory volume in 1 s
HNPs: Human neutrophil peptides
HRCT: High-resolution computed tomography
IL-8: Interleukin-8
LCI: Lung clearance index
LTB4: Leukotriene-B4
MAC: Mycobacterium avium complex
NE: Neutrophil elastase
PBB: Protracted bacterial bronchitis
PCD: Primary ciliary dyskinesia
PFT: Pulmonary function tests
PI: Pancreas insufficiency
ROS: Reactive oxygen species
